# Role of Th17 cells, Treg cells, and Th17/Treg imbalance in immune homeostasis disorders in patients with chronic obstructive pulmonary disease

**DOI:** 10.1002/iid3.784

**Published:** 2023-02-24

**Authors:** Ru Ma, Hongling Su, Keping Jiao, Jian Liu

**Affiliations:** ^1^ Department of The First Clinical School of Medicine Lanzhou University Lanzhou China; ^2^ Department of Gansu Provincial People's Hospital Lanzhou China

**Keywords:** chronic obstructive pulmonary disease, immune system, T helper cells, T‐regulatory cells

## Abstract

Chronic obstructive pulmonary disease (COPD) is the third leading cause of death worldwide, following strokes and cardiovascular diseases. Chronic lung inflammation is believed to play a role in the development of COPD. In addition, accumulating evidence shows that the immune system plays a crucial role in the pathogenesis of COPD. Significant advancements have been made in research on the pathogenesis of immune diseases and chronic inflammation in recent years, and T helper 17 (Th17) cells and regulatory T (Treg) cells have been found to play a crucial role in the autoimmune response. Th17 cells are a proinflammatory subpopulation that causes autoimmune disease and tissue damage. Treg cells, on the other hand, have a negative effect but can contribute to the occurrence of the same disease when their antagonism fails. This review mainly summarizes the biological characteristics of Th17 cells and Treg cells, their roles in chronic inflammatory diseases of COPD, and the role of the Th17/Treg ratio in the onset, development, and outcome of inflammatory disorders, as well as recent advancements in immunomodulatory treatment targeting Th17/Treg cells in COPD.

## Graphical abstract

Disturbed immune homeostasis in patients with chronic obstructive pulmonary disease. There was an increase in the number and function of T helper 17 cells and their associated cytokines and, conversely, a decrease in the number and function of regulatory T cells and their associated cytokines.

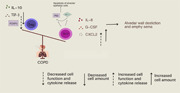

## 1 INTRODUCTION

Chronic obstructive pulmonary disease (COPD) is an inflammatory disease caused by airway and/or alveolar abnormalities that results in chronic airflow limitation and respiratory problems.^1^ This irreversible airflow obstruction further results in high morbidity and mortality.^2^ According to the World Health Organization, COPD will remain the third leading cause of mortality until 2030.^3^ Additionally, the finding of genetic risk factors for various COPD subtypes implies that the condition is a spectrum with numerous forms, each with its own unique histopathological characteristics.^4^, ^5^, ^6^ As a result, any incomplete combustion of organic matter, in combination with its own inherited susceptibility, initiates and propagates an immunological response, resulting in a variety of COPD‐related symptoms.^7^

The immune system responds to environmental stimuli and strives to protect the host by killing rogue cells like cancer, neutralizing potential invaders like infections, and organizing tertiary immune structures in mucosal tissues like the airways.^8^, ^9^, ^10^ T‐cell‐mediated adaptive immunity plays a significant role in controlling airway inflammation.^11^ CD4^+^ T cells are divided into two subsets: T helper 17 (Th17) and regulatory T (Treg) cells, which differ in development and function. The maintenance of the balance of these cells is crucial for controlling the body's immune conditions, and an imbalance can cause systemic or local abnormal immune responses. In this article, we will concisely summarize the function of Th17 cells and Treg cells, as well as evidence of their involvement in the pathogenesis of COPD. We also anticipate their future application in COPD management.

## 2 TH17 CELLS

### 2.1 Overview of Th cells

Lung nesting CD4^+^ T lymphocytes distinguish secreted cytokines as Th1 (interleukin‐2 [IL‐2] and interferon‐γ [IFN‐γ] secretion), Th2 (IL‐4, IL‐5, and IL‐13 secretion), or Th17 (IL‐17a, IL‐17f, and IL‐22 secretion) subpopulations.^12^, ^13^, ^14^

In animal experiments, Th17 cells have two different upstream signal transduction pathways due to their different differentiation sources. The first upstream pathway includes IL‐6 and transforming growth factor‐β (TGF‐β), both of which costimulate Th0 cells. The combination of IL‐6 and IL‐6R stimulates signal transducer and activator of transcription 3 (STAT3), and the combination of TGF‐β and TGF receptor activates the phosphorylation of bone morphogenetic protein‐related proteins (Smads), which triggers IL‐17 and the transcription factor retinoic acid‐related orphan receptor‐γ (RORt) to differentiate Th17 cells.^15^ In addition, IL‐21 plays a crucial role in Th17 elaboration and differentiation. Although not as effective as IL‐6, TGF‐β and IL‐21 can also cause differentiation of Th cells in the absence of IL‐6.^16^, ^17^, ^18^ A second upstream pathway includes the secretion of IL‐21 by natural killer cells following TGF‐β binding. In the presence of IL‐17, IL‐21 stimulates the production of STAT‐3 and RORt, while TGF‐β upregulates RORt expression to secrete IL‐17, which works on transcription factor RORt and provides positive feedback to Th17 cells. The autocrine IL‐21 and STAT3 effects of Th17 cells are fed back to Th17 cells to form an IL‐21 autocrine loop and enhance the expression of IL‐23 and RORγt, which further promotes the increase in the number of Th17 and presents an amplifying effect.^19^ Early cell proliferation can be induced by the primary components of the autocrine loop of Th17 cells, TGF‐β and IL‐21.^16^, ^17^, ^18^ Furthermore, the downstream activity includes members of the IL‐23 and IL‐21 cytokine families stimulated in Th17 after the initiation and expansion of differentiation and maintaining phenotypic stability. However, since natural T cells do not put IL‐23 receptors, this cytokine alone cannot induce naive T cells to differentiate into Th17 cells.^20^ TGF‐β and IL‐6 prompt the expression of IL‐23 receptors on Th17 cells, and these cells are also sensitive to IL‐23,^16^, ^17^, ^18^, ^21^ resulting in upregulation of matrix metalloproteinase 9 (MMP9). In short, Th17 differentiation includes three measures, TGF‐β and IL‐6 promote its differentiation, IL‐21 assists in its amplification, and IL‐23 maintains its stability.^22^

In humans, Th17 cells are copied from the CD161^+^CD4^+^ T‐cell subsets of the thymus, which differentiate into Th17 cells expressing RORγt and IL‐23R in the presence of IL‐1β and IL‐23 and maintain their survival. Moreover, the development of human Th17 cells from naive T cells requires TGF‐β.^23^, ^24^ In summary, human IL‐1β and IL‐23 are complicated in Th17 cell differentiation, and Th17 cells autocrinely produce IL‐21 to expand the scale of separation, activate STAT3, and induce the expression of RORγt. Although IL‐23 does not initially promote Th17 differentiation, it is crucial for T‐cell survival, growth, and maintenance.^25^

### 2.2 Th17 cells and COPD

Th17 cells play a significant role in the systemic inflammation associated with COPD. Activated Th17 cells can produce inflammatory cytokines such as IL‐17 to stimulate the Janus kinase/STAT signaling cascade.^26^ In addition, differentiated Th17 cells directly stimulate the activation of the RANK/RANKL signaling pathway to enhance RANKL expression. This pathway is involved in mediating cigarette smoke‐induced increases in alveolar macrophage MMP‐9 expression, which leads to increased airway inflammation and emphysema.^27^ Additionally, RANK was found to be involved in IL‐17A‐dependent lymphoid neogenesis, which might shed light on the molecular basis of smoking‐related pulmonary lymphoid neogenesis in COPD patients.^28^ At present, most studies on the role of Th17 cells in the pathogenesis of COPD have focused on the expression and impact of IL‐17 in the airway, with IL‐17A being the most identified in the IL‐17 family and most comparable to IL‐17F.

**Figure 1:**
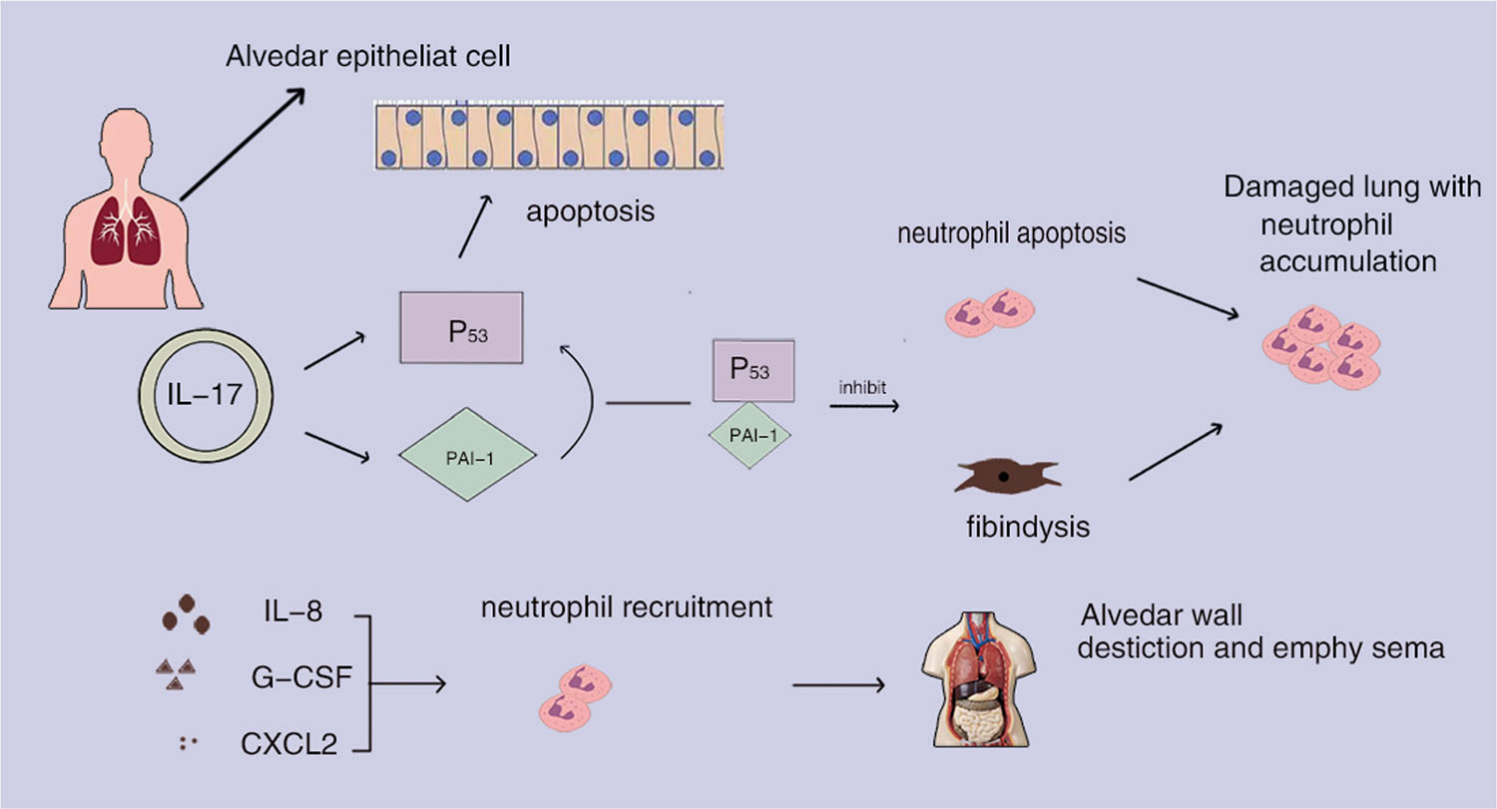
By raising p53 and plasminogen activator inhibitor‐1 (PAI‐1), interleukin‐17A (IL‐17A) encourages neutrophil infiltration and lung damage. Alveolar epithelial cells can undergo apoptosis when exposed to both p53 and PAI‐1; however, PAI‐1 prevents neutrophil apoptosis and fibrinolysis in lung tissue. Additionally, IL‐17A stimulates the production of IL‐8, granulocyte‐colony stimulating factor (G‐CSF), and C–X–C motif chemokine ligand 2 (CXCL2), which draws in neutrophils and causes them to produce neutrophil elastase and myeloperoxidase, leading to the breakdown of the alveolar wall and the development of emphysema.

IL‐17A has been found to facilitate neutrophil inflammation in COPD patients. COPD (18 cases, average age 72 years, 13 male, FEV1% = 63.5% [FEV1% is first second expiratory volume as a percentage of expiratory lung volume]) increased numerous IL‐17‐expressing inflammatory cells in the small airway epithelium compared to smokers and nonsmokers.^29^ Smokers with COPD showed higher levels of IL‐17A, p53, and plasminogen activator inhibitor‐1 (PAI‐1) than healthy smokers (HSs) and healthy control subjects (HCs).^30^ The upregulation of IL‐17A promoted alveolar basal epithelial cell motility and raised p53 and PAI‐1 production in the experiment using bleomycin‐induced alveolar basal epithelial cells to simulate the inflammation in vitro.^31^ Furthermore, IL‐17 can increase the expression of p53 and PAI‐1, as well as increase C–X–C motif chemokine ligand 1 (CXCL1), CXCL2, and C–X–C chemokine receptor 2 to induce neutrophil influx, hence enhancing the neutrophil inflammatory reaction in COPD. IL‐17 can also induce the expression of neutrophil chemokines IL‐8, granulocyte‐colony stimulating factor, and CXCL2, and recruit neutrophils to reach inflammatory lesions.^32^ A rise in neutrophils could release more myeloperoxidase and neutrophil elastase (NE), which can erode collagen, weaken alveolar walls, and result in emphysema^33^ (Figure 1).

Meanwhile, IL‐17A was also found to promote airway remodeling in COPD. COPD‐related lung structural remodeling can result in permanent airflow obstruction.^34^ In animal models of pneumonia and fibrosis, IL‐17A modulates collagen synthesis and secretion in alveolar epithelial cells and promotes epithelial‐to‐mesenchymal transition, while also inhibiting autophagy and promoting autophagy‐related cell death in inflamed lung tissue in a TGF‐dependent manner.^35^, ^36^, ^37^, ^38^, ^39^ Because collagen and inflammation‐related mediators cannot be degraded by autophagy, this results in more severe inflammation and airway remodeling.^40^ There have been claims that IL‐17A is associated with histone deacetylase 2 (HDAC2) on airway remodeling and collagen deposition in COPD, with contrary effects. While IL‐17A exacerbates CS‐induced airway remodeling and causes inflammatory cells to release the profibrotic cytokine TGF‐1, HDACs prevent the development of Th17 cells by reversing the hyperacetylation of core histones. As a result, the less IL‐17 produced by Th17 cells, the more attenuated or even inhibited airway remodeling in COPD patients.^41^ Taken together, IL‐17 can stimulate airway remodeling by increasing TGF‐1 and suppressing inflammatory mediators and autophagy in injured lung tissue.

### 2.3 Targeting key mediators of inflammation: IL‐17A

Evidence from preclinical and clinical trials demonstrates the role of adaptive immunity in the progression of COPD. Only CD4^+^ Th17 cells from the T‐lymphocyte lineage have been found to express IL‐17A. Other experimental studies have revealed the existence of numerous IL‐17a induction pathways, including bone‐bridging proteins and other proinflammatory mediators. These are primarily induced by CD1a^+^ conventional dendritic cells, primary lung antigen‐presenting cells (APCs) that promote Th17 cell differentiation through the action of a secreted cytokine, osteopontin (Spp1).^42^ In line with other preclinical indicators, neutral inhibition of IL‐17a and bone‐bridging proteins are tested at the prophylactic level. Therefore, the question of how effectively inhibition of IL‐17a affects COPD progression remains a clinical question. The clinical practice uses commercially accessible, Food and Drug Administration‐approved anti‐TNF, anti‐IL‐6, and anti‐IL‐17A biologics to treat autoimmunity; as predicted, these treatments eventually raise the risk of infection. These treatment options may potentially increase the risk of COPD exacerbation. Abrogating adaptive (memory) immune cells against smokers' self‐antigens is hence the suggested line of action. Studies on Treg cells are required, for instance, to look into the possibility of enhancing Treg cell subsets or, alternatively, the removal of autoreactive T cells utilizing cutting‐edge cellular therapies like chimeric antigen receptor T cells.

## 3 TREG CELLS

### 3.1 Development, differentiation, functional mechanism, and phenotypic characteristics of Treg cells

In the thymic selection process, T lymphocytes with high affinities for self‐proteins are removed, whereas those with low affinities can differentiate into Treg cells, a specialized subgroup of CD4^+^ T cells (Treg cells). These cells block T‐lymphocyte activation by expressing the canonical transcription factor forkhead box P3 (Foxp3).^43^, ^44^, ^45^ In addition to immunosuppressive effects, Treg cells can also maintain tissue balance. For instance, amphiregulin released by Treg cells promotes tissue repair in addition to its well‐known immunoregulatory role.^46^, ^47^ Research has shown that the proliferation of Th cells mainly depends on glycolytic metabolic pathways. The inhibitory function of Treg cells is more dependent on the mitochondrial oxidation pathway. Gerriets et al.^48^ found that activating the PI3K–AKT–mTORC1 axis in Treg cell makes glycolysis promote the proliferation of Treg cell, and the toll‐like receptor signaling route activates the axis.

Treg cells protect themselves against overactivated immune responses. The reduction in the number of Treg cells is linked to the dysfunction of many immune diseases.^49^ Therefore, understanding the mechanism of Treg cell function is critical. Studies have found that Treg cells are activated and release granzyme A and perforin to kill effector cells or APCs.^50^ or express the costimulatory molecules CTLA‐4 and CD80 and/or CD86 on dendritic cells to regulate them.^51^, ^52^ Treg cells can also function through mediator‐mediated mechanisms. Immunomodulatory molecules such IL‐35, IL‐10, TGF‐β, and lymphocyte‐activation gene 3 are critically involved in Treg cell function, according to studies.^53^, ^54^, ^55^ Akkaya et al.^56^ confirmed that the antigen‐specific Treg cells can downregulate the antigen presentation function of DCs by reducing the peptide–major histocompatibility complex class II on the surface of DCs. Treg cells regulate the function of DCs by microRNA transfer from Tregs to DCs, especially miR‐150‐5p and miR‐142‐3p,^57^ which can upregulate IL‐10 and downregulate IL‐6 production.^58^

### 3.2 Treg cells and COPD

Treg cells are marked by the expression of Foxp3 and CD25, which inhibit the proliferation of other T cells, as well as the release of anti‐inflammatory cytokines such as IL‐10 and TGF‐β, which are essential for immune tolerance and immune homeostasis.^59^, ^60^ Moreover, Treg cells play an important role in respiratory viral infections by suppressing hyperstimulated inflammatory responses and tissue damage induced by other innate and adaptive immune elements.^61^ Treg cells may contribute to the onset of autoimmune disorders; however, research on their function in COPD is ongoing.

In patients with acute COPD, the number of Treg cells is higher, and the number of Treg cells in patients with stable COPD and normal people is lower. It shows that patients with acute COPD are greatly affected by Treg cells. When inflammation develops more severely, negative feedback immune regulation can cause Treg cells to inhibit the inflammatory response and reduce the severity of the disease.^62^ Certain microenvironments may be highly helpful for the differentiation of Treg cells in the acute phase of COPD, which is positively connected with the severity of the disease.^63^

**Figure 2:**
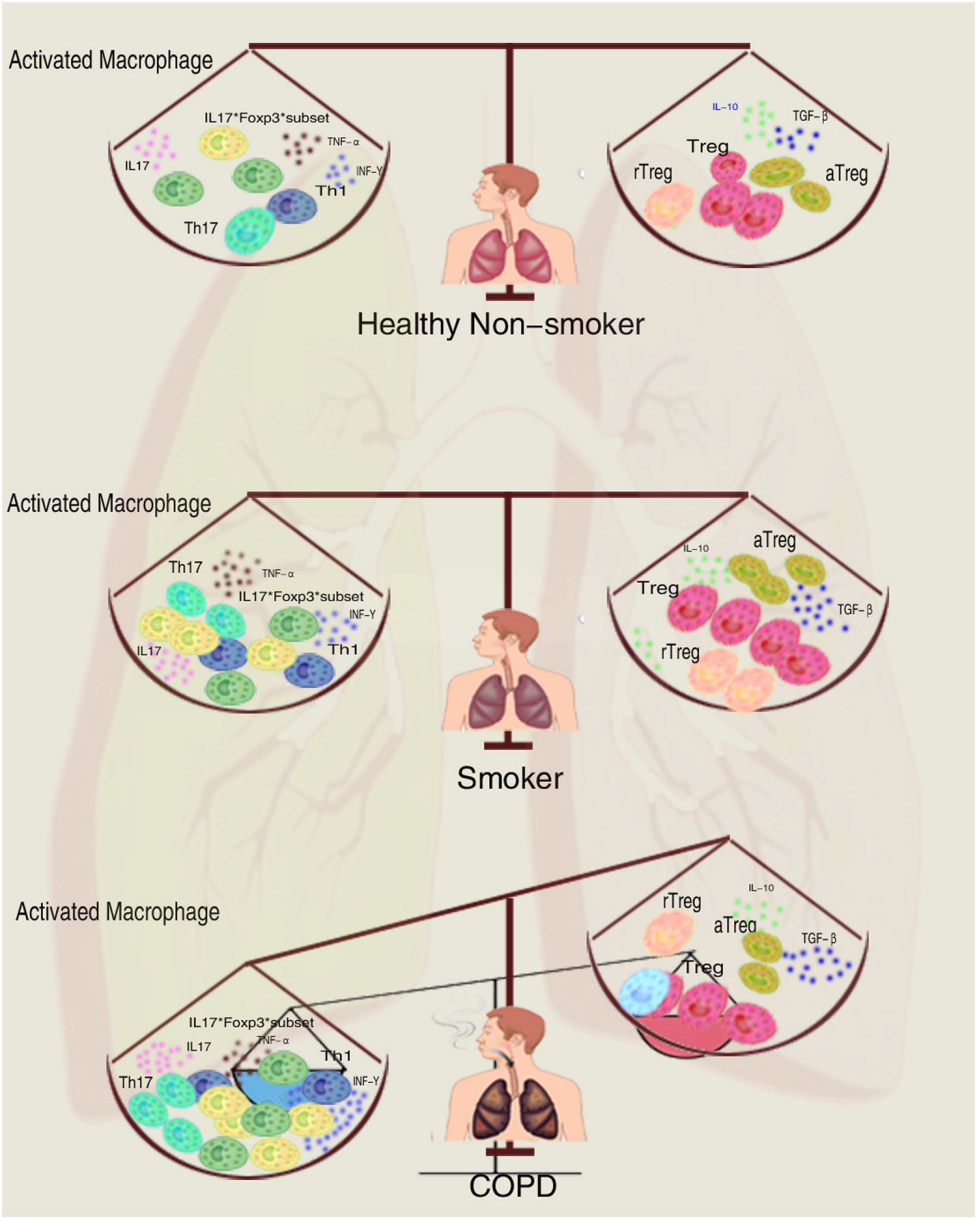
Immune homeostasis disturbance in chronic obstructive pulmonary disease (COPD) patients. Smoking causes a proinflammatory response, but the compensatory anti‐inflammatory system maintains immunological homeostasis in smokers just as it does in healthy nonsmokers. Long‐term smoking can exhaust the anti‐inflammatory compensatory capacity in COPD patients, which causes an imbalance between pro‐ and anti‐inflammatory pathways and eventually disturbs immunological homeostasis. aTreg, activated regulatory T cell; Foxp3, forkhead box P3; IL, interleukin; rTreg, resting Treg; TGF‐β, transforming growth factor‐β; Th, T helper.

The amount of Treg cells in BAL fluid and different lung tissues vary in people with COPD. Patients with moderate COPD had more CD4^+^Foxp3^+^ T cells in lung lymphocyte follicles and no CD4^+^Foxp3^+^ T cells in the pulmonary parenchyma.^64^ Studies have shown that Foxp3^+^ cells are upregulated in the large airways and downregulated in the tiny airways. Recent studies have demonstrated that CD4+CD25+Foxp3+ T cells can develop into proinflammatory Th17 cells, which may contribute to the persistence of COPD‐related chronic inflammation.^65^ Inferring from the function of Treg cells, there are data suggesting that smokers with normal lung function may increase Treg cells, while COPD patients should decrease Treg cells. Damage to Treg cells may destroy the homeostasis of the body, resulting in persistent lung inflammation, accompanied by severe lung injury and COPD (Figure 2). This immunological equilibrium exists between several functional subsets of Treg cells and inflammatory cells, such as Th17, Th1, and CD8^+^ T cells. Given that little is known about the cellular plasticity of Treg cells in vivo, targeting them in the lung parenchyma is a considerable challenge. Furthermore, cytokines like TGF‐β, which are essential for Treg polarization, can also cause fibrosis, and the plasticity of inducible Treg cells in the presence of IL‐6, which promotes a pathogenic FOXP3^+^IL‐17A^+^ double‐positive population in vivo, are also known to have these effects.^66^, ^67^ Therapeutic studies on Treg cells have been shown to be effective in other models of autoimmune disease.^68^, ^69^ Increased IL‐10, for example, can promote cytostatic action and control inflammation in the lungs.

## 4 TH17/TREG IMBALANCE IN COPD

Two CD4^+^ T‐cell subpopulations (Th17 and Treg cells) have opposing roles in the immunopathogenesis of autoimmune and chronic inflammatory diseases. Th17 cells, as proinflammatory effector T cells, promote autoimmunity, although Treg cells can help by maintaining self‐tolerance and controlling autoimmune responses. Dysregulation of the Th17/Treg cell balance has been linked to the onset and development of various diseases, including cancer, inflammatory diseases, and autoimmune diseases. A variety of factors, including T‐cell receptor (TCR) signaling, cytokines, and metabolic and epigenetic regulators, can influence the differentiation of Th17 and Treg cells and affect their homeostasis. Increasing evidence suggests that the number of posttranslational modifications (PTMs), including phosphorylation, methylation, nitrosylation, acetylation, glycosylation, lipidation, ubiquitination, and SUMOylation, regulates the activity of important molecules, including Foxp3, RORt, and STAT.^69^ In addition, PTMs may influence protein folding efficiency and conformational stability, which affects protein structure, location, and function, and hence influences the balance of Th17 and Treg cells. Hence, Th17/Treg cells are believed to be involved in the pathogenesis and disease progression of autoimmune diseases like COPD.

In tissue from COPD Stage I and Stage II patients, Zheng et al.^70^ examined the imbalance of Th17/Treg cells and compared it to HSs and nonsmoking control people. Both immunohistochemical and flow cytometry measurements of the COPD group revealed a gradual rise in Th17 cells and a fall in Treg cells.^70^ Th17/Treg imbalance in tissue samples from COPD subjects was also found in previous studies.^71^ In the present study, IL‐17^+^ cells were increased in both the COPD group and HSs, while the number of Treg (Foxp3^+^) and IL‐10^+^ cells in the small airways of smokers with obstructive lung disease compared with HSs and controls was reduced. Conversely, the authors discovered an increase in Treg cells in lymphoid tissue.^71^ Moreover, an increase in the Th17/Treg ratio was found to be negatively associated with lung function. There is an interventricular imbalance of Th17 cells relative to Treg cells in COPD patients' airways, implying a flaw in COPD anti‐inflammatory homeostasis.^72^ Another finding suggests that plasma fossa 1 plays a role in Th17/Treg balance. Loss of plasma fossa 1 causes Th17/Treg imbalance.^73^ There is also evidence that damage to the TGF‐β/BAMBI (BMP, activin, membrane‐bound inhibitor) pathway may increase the inflammatory response, resulting in a Th17/Treg imbalance.^74^

HSs, stable COPD, and acute exacerbation COPD all had more peripheral blood Th17 cells and fewer Treg cells than healthy nonsmokers,^70^ indicating an imbalance in the Th17/Treg ratio. These findings are also consistent with the lung tissue damage; however, peripheral blood mononuclear cell samples and lung tissue samples were not from the same subject.^70^ Indeed, a few research have assessed lung and peripheral blood samples obtained from the same patient,^75^, ^76^ which may be related to a limiting element when studying human samples. A study on Th17/Treg cell balance in periodontitis showed that the immune system, skeletal muscle, and microorganisms all affect the balance between Th17/Treg cells.^77^ The next step might be to determine whether the microbial environment in COPD patients can impact the balance of Th17/Treg cells and regulate their differentiation.

## 5 CONCLUSION

The pathogenesis of COPD is very complicated. The existing evidence indicates that several immune cell subgroups interact with one another to contribute to the development of COPD. This article reviews the role of Th17 and Treg cells and their associated cytokines in the pathogenesis and progression of COPD, as well as the latest research on their role in COPD diagnosis and management. The immune pathogenesis of COPD chronic injury is closely connected to the continuous airway inflammation mediated by CD4^+^ T cells and the changes in the serum cytokine microenvironment. At the same time, the normal balance between Th17 and Treg cells in COPD patients was disrupted. Patients with acute exacerbations of COPD showed a proinflammatory response, while patients with stable COPD showed an anti‐inflammatory response. Since the Th17/Treg imbalance is essential in the COPD lung immune response, the Th17/Treg ratio can be utilized to predict COPD progression. Therefore, specific targeting of Th17/Treg effector cell regulation and/or cytokines that promote their differentiation may provide new strategies for COPD prevention, management, and treatment. Overall, numerous lines of evidence show that T lymphocytes are increased in the lungs of emphysema patients. These findings also raise several questions: How do CD8 and CD4 T lymphocytes differentially contribute to COPD? How do T lymphocytes interact with other immune and nonimmune cells in the lung and how the interactions may differ in disease and homeostasis? The most intriguing question is perhaps why there is augmented TCR signaling in COPD? What triggers and sustains this TCR activation? Not only that but it is also crucial to differentiate between Treg cell populations with different phenotypes and functions to better understand their immunosuppressive activity in COPD. Suppression of adaptive immunity in patients with COPD does not lead to optimal clinical care. While a strong innate immunity is required as the first line of defence against respiratory pathogens, effective eradication of viral, bacterial, or fungal pathogens requires an adaptive immune response. There are still many challenges in the pathogenesis of cigarette smoke‐induced COPD, and we need to develop more effective animal models that realistically replicate the natural history, pathological features, and comorbidities of COPD in humans, as well as explore new treatment approaches.

## AUTHOR CONTRIBUTIONS

**Ru Ma**: Data curation; investigation; methodology; writing—original draft. **Kongling Su and Keping Jiao**: Data acquisition and interpretation. **Jian Liu**: Review and editing; supervision; validation.

## CONFLICT OF INTEREST STATEMENT

The authors declare no conflict of interest.

## Data Availability

All data generated or analyzed during this study are included in this article.
